# Elevated plasma fatty acid-binding protein 3 is related to prolonged corrected QT interval and reduced ejection fraction in patients with stable angina

**DOI:** 10.7150/ijms.54508

**Published:** 2021-03-11

**Authors:** Yung-Chuan Lu, Thung-Lip Lee, Chin-Feng Hsuan, Wei-Chin Hung, Cheng-Ching Wu, Chao-Ping Wang, Ching-Ting Wei, Teng-Hung Yu, Fu-Mei Chung, Yau-Jiunn Lee, I-Ting Tsai

**Affiliations:** 1 Division of Endocrinology and Metabolism, Department of Internal Medicine, E-Da Hospital, Kaohsiung, 82445 Taiwan.; 2 Division of Cardiology, Department of Internal Medicine, E-Da Hospital, Kaohsiung, 82445 Taiwan.; 3 Division of General Surgery, Department of Surgery, E-Da Hospital, Kaohsiung, 82445 Taiwan.; 4 Department of Emergency, E-Da Hospital, Kaohsiung, 82445 Taiwan.; 5 Lee's Endocrinology Clinic, Pingtung, 90000 Taiwan.; 6 School of Medicine, College of Medicine, I-Shou University, Kaohsiung, 82445 Taiwan.; 7 The School of Chinese Medicine for Post Baccalaureate, College of Medicine, I-Shou University, Kaohsiung, 82445 Taiwan.; 8 School of Medicine for International Students, College of Medicine, I-Shou University, Kaohsiung, 82445 Taiwan.; 9 Department of Biomedical Engineering, I-Shou University, Kaohsiung, 82445 Taiwan.; 10 Department of Electrical Engineering, I-Shou University, Kaohsiung, 82445 Taiwan.; 11 Division of Cardiology, Department of Internal Medicine, E-Da Cancer Hospital, Kaohsiung 82445 Taiwan.; 12 Division of Cardiology, Department of Internal Medicine, E-Da Dachang Hospital, Kaohsiung, Taiwan.

**Keywords:** fatty acid-binding protein 3, corrected QT Interval, ejection fraction, inflammation, stable angina

## Abstract

**Background:** Higher concentrations of plasma fatty acid-binding protein 3 (FABP3) play a role in the development of cardiovascular events, cerebrovascular deaths, and acute heart failure. However, little is known about the relationship between plasma FABP3 level and prolonged QT interval and reduced ejection fraction (EF). This study aimed to investigate the relationship between plasma FABP3 level and prolonged corrected QT (QTc) interval and reduced EF in patients with stable angina. Inflammatory cytokine and adipocytokine levels were also measured to investigate their associations with plasma FABP3.

**Methods:** We evaluated 249 consecutive patients with stable angina. Circulating levels of FABP3 were measured by ELISA. In addition, 12-lead ECG and echocardiography recordings were obtained from each patient.

**Results:** Multiple regression analysis showed that high-density lipoprotein cholesterol, high sensitivity C-reactive protein (hs-CRP), white blood cell (WBC) count, visfatin, adiponectin, FABP4, heart rate, QTc interval, left atrial diameter, left ventricular mass index, end-systolic volume, end-systolic volume index, fractional shortening, and EF were independently associated with FABP3 (all *p*<0.05). Patients with an abnormal QTc interval had a higher median plasma FABP3 level than those with a borderline and normal QTc interval. With increasing FABP3 tertiles, the patients had higher frequencies of abnormal QTc interval, left ventricular systolic dysfunction, and all-cause mortality, incrementally lower EF, higher WBC count, and higher levels of hs-CRP, visfatin, adiponectin, and FABP4.

**Conclusion:** This study indicates that plasma FABP3 may act as a surrogate parameter of prolonged QTc interval and reduced EF in patients with stable angina, partially through the effects of inflammation or cardiomyocyte injury. Further studies are required to elucidate whether plasma FABP3 plays a role in the pathogenesis of QTc prolongation and reduced EF.

## Introduction

Cardiovascular disease (CVD) is one of the main causes of death in most countries 1 and includes related heart and blood vessel problems such as coronary heart disease, heart failure, congenital heart disease, raised blood pressure, peripheral artery disease, rheumatic heart disease, and cerebrovascular disease 2. An estimated 25 million new cases of heart disease are diagnosed each year 3, and thus identifying the risk factors for CVD is of crucial importance to improve long-term therapeutic outcomes and prevent and reduce wasting medical resources.

Chronic heart failure (CHF) is one of the most common cardiovascular disorders. It is associated with unfavorable outcomes and often coexists with other chronic diseases that further affect the prognosis 4,5. Among a variety of parameters reflecting disease severity, left ventricular ejection fraction (LVEF) remains one of the strongest long-term prognostic factors.

Prolonged QTc interval and reduced EF are complex traits which can be affected by both genetic and environmental factors 6,7. Previous studies have shown that during reperfusion of ischemic myocardium, cytokines such as platelet-activating factor generated by activated neutrophils can cause arrhythmia 8,9. Furthermore, Chung et al. showed that elevated C-reactive protein (CRP) may reflect an inflammatory state that promotes the persistence of atrial fibrillation (AF) 10. Moreover, Arroyo- Espliguero et al. demonstrated an inverse correlation between high-sensitive (hs)-CRP serum concentration and LVEF, and that hs-CRP was an independent predictor of NYHA functional class in patients with chronic stable angina 11. Moreover, increased levels of inflammatory mediators in patients with stable angina have been associated with cardiac fibrosis, arrhythmias, and CHF 8,9,12, resulting in a higher risk of these disorders and even death.

Fatty acid-binding protein 3 (FABP3) (also known as heart-type fatty acid-binding protein or H-FABP) is a type of intra-cardiac protein that plays an important role in the metabolism of fatty acids inside cardiomyocytes 13. FABP3 is mainly found in the heart and has a molecular weight of 15 kDa. When there is an ischemic injury to the myocardium, FABP3 is rapidly released into the circulatory system and is then eliminated by the kidneys. Consequently, serum FABP3 measurements can reflect myocardial injury. Furthermore, FABP3 has been associated with other clinical parameters in patients with acute myocardial infarction (AMI) such as EF, length of hospital stay, high-sensitive troponin T and N-terminal pro-brain natriuretic peptide levels as well as inflammatory markers (CRP, leucocytes) 14. Moreover, FABP3 is not only a sensitive marker of myocardial injury when it is over-expressed, but it has also been shown to promote growth, inflammation, and migration of vascular smooth muscle cells, and thus it could potentially play a role in the pathophysiology of in-stent restenosis 15. Given the association between QT interval prolongation, reduced EF, coronary artery disease (CAD), and inflammation, and given the inflammatory characteristics of a high plasma FABP3 level and its relationship with CVD, we hypothesized that FABP3 may be a surrogate marker independently associated with QTc interval prolongation and reduced EF in humans.

To the best of our knowledge, little is known about the relationship between FABP3 level and cardiac electro-pathology and LVEF. Therefore, the aim of this study was to investigate whether FABP3 levels were associated with a prolonged QTc interval and reduced EF in a cohort of patients with stable angina. Inflammatory cytokine and adipocytokine levels were also measured to investigate their associations with plasma FABP3.

## Methods

### Study participants

We prospectively screened consecutive patients referred for percutaneous coronary interventions (PCIs) from July 2007 to July 2015 at E-Da Hospital. We included patients with both stable angina pectoris and successful PCI. Stable angina pectoris was diagnosed by the cardiologist in charge as previously described 16,17. Successful PCI was defined as a final angiographic residual stenosis of <30% as determined by quantitative coronary angiography without flow-limiting dissection or occlusion of the large branch (>1 mm) and a resulting Thrombolysis in Myocardial Infarction grade 3 18. The exclusion criteria were patients who experienced periprocedural myocardial injury, defined as an increase in troponin T three times greater than the upper limit of the reference range 24 hours after PCI 19, patients with a history of acute coronary syndrome (ACS) and psychosis, inflammatory diseases (including infection or sepsis), collagen diseases, liver diseases, malignancy, steroid use, and those with a bundle branch block pattern. In addition, patients with moderate to severe valvular heart disease diagnosed according to a previous report 20, patients taking medications which may have influenced the QT interval including psychotropic medications and class I (e.g. mexiletine, flecainide, quinidine, and procainamide) and class III (e.g. amiodarone, dronedarone, and vernakalant) anti-arrhythmic medications were also excluded from the study. Written informed consent was obtained from the patients before enrollment. The study followed the guidelines of and was approved by the Human Research Ethics Committee at our institution.

Before the coronary angiography examinations, detailed records of each patient with regards to personal and medical histories were reviewed. The following CVD risk factors were assessed. Patients with a current or prior diagnosis of type 2 diabetes and those receiving medical therapy for type 2 diabetes were defined as having type 2 diabetes in accordance with the World Health Organization guidelines 21. Arterial hypertension was diagnosed in patients with resting blood pressure values ≥140/90 mmHg, and in patients with a history of hypertension and taking anti-hypertensive drugs. Hyperlipidemia was defined as a fasting total serum cholesterol level >200 mg/dL, low-density lipoprotein cholesterol (LDL-C) level >130 mg/dL, high-density lipoprotein cholesterol (HDL-C) level <40 mg/dl or serum triglyceride level >180 mg/dL; moreover, all patients receiving lipid-lowering therapy, which was routinely administered at our institution during the study period, were diagnosed with hyperlipidemia. Smoking status was classified as non-smoker, former smoker (having stopped smoking for ≥1 year), or current smoker. Current and former smokers were grouped for analysis and compared to the never smokers.

### Laboratory measurements

Before the coronary angiography examinations, plasma biochemical parameters were measured in all of the patients after fasting for 8 hours. All biochemical analyses were performed at the E-Da Hospital laboratory within 2 hours of the blood samples being drawn. Serum triglycerides, total cholesterol, LDL-C, HDL-C, albumin, glucose, and white blood cell (WBC) count were determined using standard commercial methods on a parallel-multichannel analyzer (SYNCHRON, Los Angeles, CA). Hemoglobin A1c (HbA1c) was measured using high performance liquid chromatography. Serum creatinine was analysed according to the kinetic Jaffé method on a SYNCHRON CX System analyzer (SYNCHRON, Los Angeles, CA) using reagents from Beckman (Beckman Coulter Diagnostic, Los Angeles, CA). In addition, the concentrations of plasma FABP3 and FABP4 were determined using enzyme-linked immunosorbent assay kits (Invitrogen, Thermo Fisher Scientific Inc., USA and R&D Systems Inc., Minneapolis, MN, USA). The dilution and standard curves were parallel, and the intra- and inter-assay coefficients of variation of the assays were 3.9% (n = 8) and 6.2% (n = 8), respectively, for FABP3, and 3.4% to 5.8% (n = 3) and 3.1% to 6.2% (n = 4), respectively, for FABP4. The concentration of plasma visfatin was determined using a commercial enzyme immunoassay kit (Phoenix Pharmaceuticals, Belmont, CA). The detection limit of this assay was 0.1 ng/mL. The serum level of high molecular weight adiponectin was determined using a commercial solid phase ELISA kit (B-Bridge International, Sunnyvale, CA). The dilution curve was parallel to the standard curve. The inter- and intra-assay coefficients of variation of the assay were 3.2-7.3% (n = 3) and 3.1-6.2% (n = 4), respectively. A high-sensitivity method was used to measure levels of plasma CRP with an IMMAGE system (Beckman Coulter, Immunochemistry Systems, Brea, CA, USA) that had a detection limit of 0.2 mg/L. The intra-assay coefficient of variation was 4.2% to 8.7% for hs-CRP. Samples were assessed in duplicate in a single experiment. Estimated glomerular filtration rates (eGFRs) were calculated using the CKD-EPI two-concentration race equation 22.

### Electrocardiogram, QT and QTc interval measurements

Twelve-lead ECG was recorded for analysis during the baseline examination using a standardized protocol when the patient was enrolled in the study. At least two cardiologists who were blinded to this study manually measured the QT interval. Full details about the electrocardiogram, QT and QTc interval measurements are provided in the supplementary materials 23-27.

### Echocardiographic examination

All patients received standard echocardiography at the follow-up visit 1 month after the PCI. Standard examinations were performed by the same experienced physician, and all of the echocardiographic measurements followed the recommendations of the American Society of Echocardiography, with at least three cycles being analyzed for each variable 28,29. Full details about the echocardiographic measurements are provided in the supplementary materials 30.

### Follow-up

The patients were reevaluated at 3, 6, and 12 months after discharge and then annually until July 2015. All of the participants received a CVD care program including a general health condition questionnaire and regular blood tests by a trained nurse to determine the occurrence of major adverse cardiovascular events and other clinical events during the entire follow-up period. If the patients could not respond to the questionnaire during regular office visits, the questionnaire was completed through personal telephone interviews. Other clinical information was obtained by reviewing hospital discharge reports relating to any other re-admission during the follow-up period. The performance of PCI or coronary artery bypass grafting (CABG) was validated by reviewing the original procedure protocols. Outcomes were adjudicated by two independent observers who were blinded with respect to the patients' baseline clinical and laboratory data.

### Statistical analysis

Data normality was analyzed using the Kolmogorov-Smirnov test. Continuous normally distributed variables are described as mean ± standard deviation, and non-normally distributed variables are described as median (interquartile range [IQR]). Statistical differences in variables were compared using one-way ANOVA for normally distributed variables followed by Tukey's pairwise comparison. Categorical variables are presented as frequencies and percentages, and inter-group comparisons were tested using the chi-square test. Logarithmically transformed values of plasma FABP3, FABP4, visfatin, hs-CRP, adiponectin, and triglycerides were used in the statistical analysis since their distributions were skewed. Simple and multiple linear regression analyses were used to examine the associations and independence between plasma FABP3 level and values of the other parameters. Statistical significance was accepted if *p* <0.05. All data were analyzed using JMP version 10.0 for Windows (SAS Institute, Cary, NC, USA).

## Results

Table 1 shows the clinical and demographic characteristics of the 249 patients with stable angina (males, 74.3%; females, 25.7%). The mean FABP3 level was 3.5 ng/mL, and the median plasma FABP3 level was 1.3 ng/mL (IQR, 0.9 ng/mL to 3.1 ng/mL). The prevalence rates of hypertension, hyperlipidemia, diabetes mellitus, left ventricular systolic dysfunction (LVSD), and abnormal QTc interval were 79.5%, 66.7%, 47.8%, 16.3%, and 31.7%, respectively. The mean QTc interval was 442 ± 37 ms, and the mean EF was 61.8 ± 12.6%. The mean follow-up period was 41.3 months (range 5 to 96 months). During the follow-up period, 109 major adverse cardiovascular events occurred in the 249 stable angina patients, of which 24 (9.6%) were death from all causes.

### Association between FABP3 and other parameters

**Figure 1:**
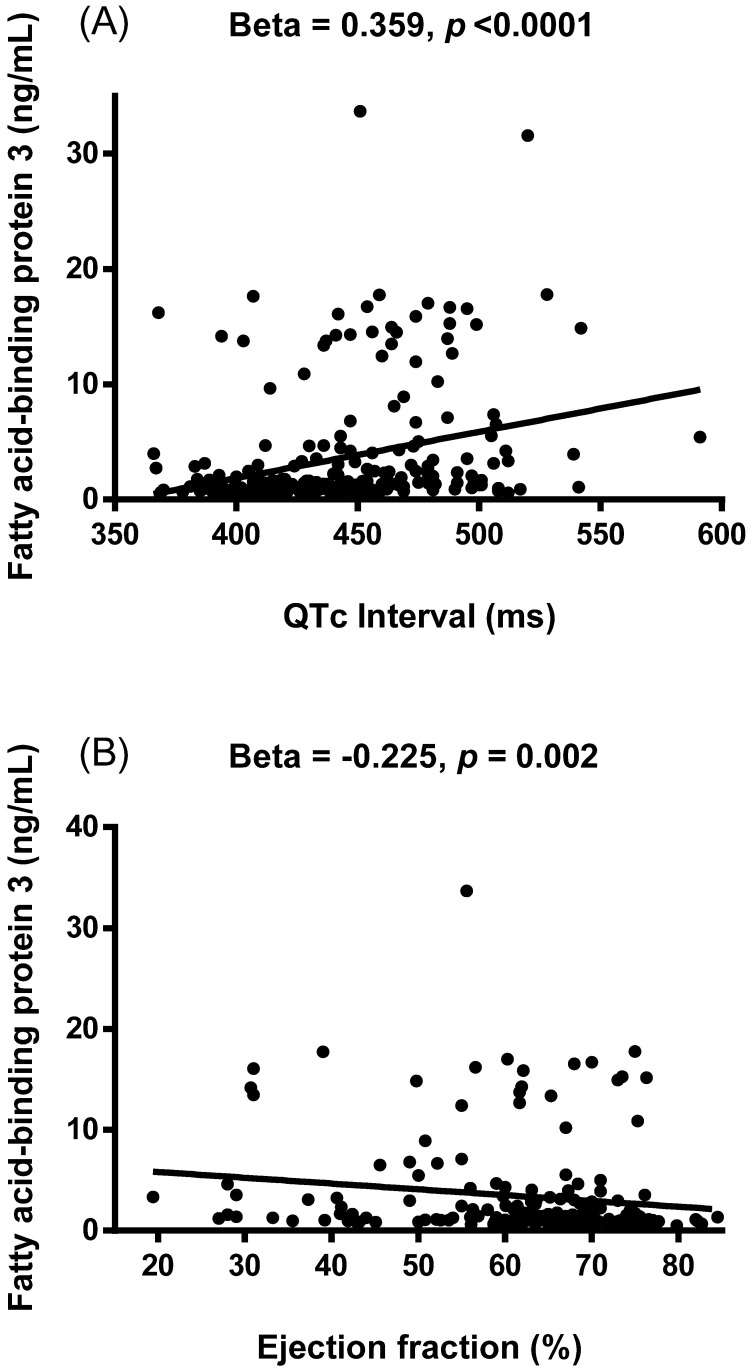
Associations between the concentration of plasma fatty acid-binding protein 3 (FABP3) and corrected QT (QTc) interval (A) and ejection fraction (B). The FABP3 concentration was positively associated with QTc interval and negatively associated with ejection fraction.

Simple linear regression analysis was performed to examine relationships between FABP3 and other variables (Table 2). The results showed that FABP3 was associated with age (β = 0.303, *p* < 0.0001), the presence of diabetes mellitus (β = 0.330, *p* < 0.0001), serum total cholesterol (β = -0.176, *p* = 0.006), HDL-C (β = -0.172, *p* = 0.007), LDL-C (β = -0.193, *p* = 0.002), albumin (β = -0.382, *p* < 0.0001), eGFR (β = -0.653, *p* < 0.0001), hs-CRP (β = 0.303, *p* < 0.0001), WBC count (β = 0.407, *p* < 0.0001), visfatin (β = 0.274, *p* = 0.030), adiponectin (β = 0.419, *p* < 0.0001), FABP4 (β = 0.769, *p* < 0.0001), heart rate (β = 0.359, *p* < 0.0001), PR interval (β = 0.175, *p* = 0.007), QTc interval (β = 0.359, *p* < 0.0001, Figure 1A), left atrial diameter (LAD) (β = 0.176, *p* = 0.017), left ventricular mass index (LVMI) (β = 0.205, *p* = 0.012), interventricular septum thickness at end-diastole (IVSd) (β = 0.138, *p* = 0.048), left ventricular posterior wall thickness at end-diastole (LVPWd) (β = 0.143, *p* = 0.041), end-systolic volume (β = 0.202, *p* = 0.010), end-systolic volume index (β = 0.234, *p* = 0.003), left ventricular internal dimension at end-systole (β = 0.179, *p* = 0.016), fractional shortening (β = -0.222, *p* = 0.001), and EF (β = -0.225, *p* = 0.002, Figure 1B).

Multiple linear regression analysis adjusted for age, sex, body mass index, diabetes mellitus, and hypertension revealed that FABP3 was positively associated with hs-CRP (β = 0.246, *p* < 0.0001), WBC count (β = 0.394, *p* < 0.0001), visfatin (β = 0.213, *p* = 0.044), adiponectin (β = 0.360, *p* < 0.0001), FABP4 (β = 0.705, *p* < 0.0001), heart rate (β = 0.283, *p* < 0.0001), QTc interval (β = 0.256, *p* < 0.0001), LAD (β = 0.183, *p* = 0.011), LVMI (β = 0.151, *p* = 0.048), end-systolic volume (β = 0.161, *p* = 0.024), and end-systolic volume index (β = 0.168, *p* = 0.022). In comparison, HDL-C (β = -0.121, *p* = 0.049), fractional shortening (β = -0.157, *p* = 0.017), and EF (β = -0.159, *p* = 0.018) were negatively associated with FABP3 (Table 3).

### Characteristics of the subjects according to the QTc prolongation status

One hundred and nineteen patients were categorized as having a normal QTc interval, compared to 51 patients with borderline QTc prolongation and 79 patients with an abnormal QTc interval (Table 4). The abnormal QTc interval group had a significantly higher plasma FABP3 level than the borderline and normal QTc interval groups (2.6 ng/mL [IQR 1.3 to 10.2] vs. 1.2 ng/mL [IQR 0.9 to 3.6] vs. 1.0 ng/mL [IQR 0.8 to 1.6], *p* < 0.0001). In addition, the patients with an abnormal QTc interval were older and had higher rates of diabetes mellitus and LVSD. Moreover, the patients with an abnormal QTc interval (prolonged QT interval) had higher levels of blood urea nitrogen, creatinine, FABP4, hs-CRP, adiponectin, and WBC count than those with a normal QTc interval. In addition, the abnormal QTc interval group had a higher fasting glucose level than the borderline and normal QTc interval groups. The patients with an abnormal QTc interval had lower levels of LDL-cholesterol, albumin, hematocrit, hemoglobin, and eGFR than those with a normal QTc interval. Furthermore, the patients with an abnormal QTc interval also had lower levels of sodium than those with a borderline QTc interval. There were no significant differences in sex, hypertension, hyperlipidemia, current smoking, BMI, waist circumference, systolic blood pressure, diastolic blood pressure, levels of potassium, calcium, HbA1c, total cholesterol, triglycerides, HDL-C, uric acid, troponin-I, number of diseased coronary arteries, Gensini score, number of stents, and receiving anti-arrhythmic medication, beta-blockers, diuretics, and statins among the three groups. Furthermore, all of the participants had stable angina with a good thrombolysis in myocardial infarction (TIMI) flow grade. As a result, there was no significant deterioration or difference between the TIMI flow grade before and after coronary angiography or angioplasty. Moreover, because all of our electrocardiographic parameters were collected before the examination, we believe that changes in TIMI flow grade would not affect our results and conclusions. In addition, when we divided the patients into groups with or without LVSD, those with LVSD had a higher level of FABP3 than those without LVSD (2.0 ng/mL [IQR 1.2 to 5.8] vs. 1.3 ng/mL [IQR 0.9 to 2.9], *p* = 0.027) (data not shown).

### Demographic and clinical characteristics according to tertiles of FABP3

Table 5 shows the patients classified according to tertiles of FABP3 as follows: low FABP3 (≤1 ng/mL), n = 81; medium FABP3 (1.1 ng/mL to 2.1 ng/mL), n = 85; and high FABP3 (>2.1 ng/mL), n = 83. The high FABP3 group had a significantly higher QTc interval than the medium and low FABP3 groups (458.5 ± 41.1 ms vs. 439.4 ± 31.7 ms vs. 427.4 ± 31.1 ms,* p* < 0.0001), and a significantly lower EF than the low FABP3 group (58.3 ± 13.6% vs. 66.1 ± 10.1%,* p* = 0.002). Furthermore, the high FABP3 group were older (*p* < 0.0001) and had higher prevalence rates of hypertension (*p* = 0.007), diabetes mellitus (*p* < 0.0001), abnormal QTc interval (*p* < 0.0001), LVSD (*p* = 0.043), and all-cause mortality (*p* < 0.0001). Moreover, the high FABP3 group had higher levels of visfatin, PR interval, LAD, LVMI, IVSd, LVPWd, end-systolic volume, end-systolic volume index, and left ventricular internal dimension at end-systole (LVID), and lower levels of total cholesterol and fractional shortening than the low FABP3 group. In addition, the high FABP3 group had higher fasting glucose, hs-CRP, WBC count, adiponectin, FABP4, and heart rate, and lower levels of LDL-C, albumin, and eGFR than the medium and low FABP3 groups.

## Discussion

In the current study, we found that plasma FABP3 levels were positively related to QTc interval in patients with stable angina and significantly increased in the patients with an abnormal QTc interval compared to those with borderline and normal QTc intervals. Furthermore, FABP3 levels were negatively related to EF, and the patients with LVSD had higher levels of FABP3 than those without LVSD. Moreover, with increasing FABP3 levels, the patients had higher rates of hypertension, diabetes mellitus, abnormal QTc interval, LVSD, and all-cause mortality, and higher levels of hs-CRP, WBC count, and visfatin. These findings are consistent with current evidence that FABP3 can be considered to be a prognostic marker for arrhythmia, heart failure, AMI, and pulmonary embolism, all of which are associated with poor clinical outcomes 31,32-34, and that inflammation induced by FABP3 may lead to CVD 15.

Previous studies have shown that AF and premature ventricular contractions can induce FABP3 leakage 35,36, and ventricular cell loss caused by apoptosis has been confirmed in AF tachycardia-induced cardiomyopathy 37,38. In addition, cardiac sympathetic nervous scintigraphy findings have shown that AF directly activates cardiac sympathetic nervous function in patients with isolated AF 39. Hence, AF-induced apoptosis and sympathetic nervous system activation may contribute to FABP3 leakage in patients with AF. Furthermore, FABP3 has been reported to be elevated in patients with Brugada syndrome and ventricular fibrillation despite the absence of structural heart disease and cardiac dysfunction 32. Therefore, assessing FABP3 could provide useful information in patients with arrhythmia. Moreover, previous studies have reported associations between proinflammatory cytokines (such as IL-6 and CRP) and arrhythmias through the modulation of ion channel function 9,40 and the aggravation of sympathetic tone 40. Myocardial fibrosis, which has been associated with inflammatory processes, can affect ventricular conduction causing a delay in repolarization that could lead to ventricular arrhythmias 40,41. Our findings suggest that inflammatory activity as reflected by hs-CRP and WBC count was a strong etiological factor in the association between higher levels of plasma FABP3 and QTc prolongation. In addition, in our previous study, we found that plasma FABP4 levels were significantly higher in patients with an abnormal QTc interval, and that they were correlated with QTc prolongation 42. In the present study, a higher FABP3 level was positively associated with plasma FABP4 level, thereby raising the possibility that FABP3 may play a role in QTc interval prolongation.

In the present study, FABP3 levels were negatively associated with EF and the patients with LVSD had higher levels of FABP3 than those without LVSD. These data are consistent with those of Arimoto et al. who reported higher serum levels of FABP3 in patients with heart failure, and that this was related to the severity of heart failure 31. During the development of cardiac hypertrophy and failure, a transition of energy substrate utilization occurs with reduced fatty acid oxidation and increased glucose utilization 43,44. Binas et al. reported that both cellular uptake and lipid oxidation of long-chain fatty acids were severely depressed in FABP3 knockout mice 45, indicating that FABP3 plays a critical role in the uptake and transport of long-chain fatty acids in cardiomyocytes. In addition, inflammation is known to play a central role in the development of heart failure 46. In this study, a higher FABP3 level was independently associated with plasma levels of hs-CRP, visfatin, and WBC count, which suggests that FABP3 may act through inflammatory responses to play an important role in the pathophysiology of reduced EF in patients with stable angina.

In this study, we also found that a high FABP3 level was significantly associated with the occurrence of all-cause mortality in patients with stable angina and that a higher FABP3 level was positively associated with the plasma level of adiponectin. In our previous study, we showed that high adiponectin plasma concentrations were independently associated with major adverse cardiovascular events in patients with CAD and type 2 diabetes mellitus 47. Suzuki et al. proposed that FABP3 could be used as a biomarker to independently predict adverse cardiac events within 30 days in patients with ACS 48. In addition, O'Donoghue et al. found that elevated FABP3 was associated with increased risks of death and other adverse cardiac events among patients with ACS, and that FABP3 alone could be used to evaluate the risk 49. Kilcullen et al. also found that FABP3 could be used to identify patients at high risk of adverse outcomes, and that proper attention should be paid to these patients to improve their prognosis 50. Furthermore, Viswanathan et al. reported that when used in combination with troponin, FABP3 measurements could help with predicting both long-term mortality and the likelihood of re-infarction in the presence of suspected ACS, even for low- to intermediate-risk subjects 51. Moreover, Agnello et al. suggested that FABP3 may be a good candidate to rule in or rule out AMI at the emergency department 52. However, in contrast, a literature review showed that FABP3 is clearly not a reliable marker of ACS, since it cannot be used to diagnose AMI, either as a stand-alone test or combined with hs-cardiac troponin 53. Further investigations are needed to clarify this issue.

The current study has some limitations. First, the number of enrolled patients was relatively small. Additional studies are necessary to prospectively examine whether a reduction in plasma FABP3 concentrations can decrease the rates of abnormal QTc interval and LVSD, and improve systolic function. Second, we enrolled individuals in whom coronary angiography was clinically indicated. This population is at an intermediate-high risk of future cardiovascular events, and our findings may not apply to lower risk individuals. Third, all of the patients' ECG parameters including QT interval and QTc were measured by the same medical technician using an identical computer-based method. As a result, we did not evaluate the variability in measured QT intervals. Fourth, whether FABP3 is associated with cardiomyocyte electrophysiology such as cardiac ion channels is still unclear, and further studies are needed to elucidate the underlying mechanisms. Furthermore, if the study population had different diseases (e.g. ACS and myocardial infarction), the diverse disease severity and condition of the study population may have impacted the results. To avoid selection bias, we chose individuals with stable angina for this study, thus the results of the present study might not be generalizable to other populations.

## Conclusions

In conclusion, elevated plasma FABP3 was closely associated with prolonged QTc Interval and reduced EF in patients with stable angina, and was independently associated with plasma levels of hs-CRP, visfatin, FABP4, and WBC count, suggesting that plasma FABP3 may act through the effects of inflammation or cardiomyocyte injury to play as a surrogate parameter of prolonged QTc Interval and reduced EF. The mechanisms by which FABP3 level and QTc interval and EF are linked remain to be investigated.

## Supplementary Material

Supplementary materials.

## Figures and Tables

**Table 1:** Clinical and demographic characteristics of the study subjects

Characteristic	Total (N = 249)
Sex (male/female)	185/64
Age (years)	71.3±11.3
Body mass index (kg/m^2^)	25.7±3.9
Waist (cm)	92.1±10.1
Hypertension (n, %)	198 (79.5)
Hyperlipidemia (n, %)	166 (66.7)
Diabetes mellitus (n, %)	119 (47.8)
Left ventricular systolic dysfunction (n, %)	30 (16.3)
Ejection fraction (%)	61.8±12.6
Abnormal QTc interval (n, %)	79 (31.7)
Major adverse cardiovascular events, n (%)	109 (43.8)
All-cause mortality (n, %)	24 (9.6)
Currently smoking (n, %)	98 (39.5)
Corrected QT interval (ms)	442±37
Systolic blood pressure (mmHg)	134±22
Diastolic blood pressure (mmHg)	76±12
Fasting glucose (mg/dl)	138.8±63.2
HbA1c (%)	6.9±1.5
T-cholesterol (mg/dl)	170.8±41.1
Triglycerides (mg/dl)	116.0 (86.0-172.0)
High-density lipoprotein-cholesterol (mg/dl)	39.7±13.3
Low-density lipoprotein -cholesterol (mg/dl)	102.1±36.1
Uric acid (mg/dl)	6.7±2.2
Creatinine (mg/dl)	1.3 (1.1-1.6)
Estimated glomerular filtration rate (ml/min/1.73m^2^)	55.5±20.3
Albumin (g/dl)	3.9±0.4
Hematocrit (%)	39.1±5.4
Hemoglobin (g/dl)	13.1±2.0
Fatty acid-binding protein 3 (ng/mL)	1.3 (0.9-3.1)
Fatty acid-binding protein 4 (ng/mL)	12.5 (6.4-21.8)
High-sensitivity C-reactive protein (mg/L)	2.3 (1.0-8.5)
Adiponectin (μg/mL)	4.6 (2.0-10.1)
White blood cell count (×10^9^/L)	7.291±2.500
Anti-arrhythmic medication (n, %)	20 (8.0)
Beta-blockers (n, %)	99 (39.8)
Diuretics (n, %)	66 (26.5)
Statins (n, %)	121 (48.6)

Data are expressed as mean ± SD, number (percentage), or median (interquartile range) for variables with a non-normal distribution.

**Table 2:** Simple linear regression analysis between fatty acid-binding protein 3 and other parameters

Parameter	Fatty acid-binding protein 3	
	β	*p*-value
Age	0.303	<0.0001
Sex	-0.083	0.194
Body mass index	0.009	0.884
Diabetes Mellitus	0.330	<0.0001
Hypertension	0.115	0.070
Hyperlipidemia	-0.104	0.101
Systolic blood pressure	0.070	0.271
Diastolic blood pressure	-0.029	0.648
Total cholesterol	-0.176	0.006
High-density lipoprotein cholesterol	-0.172	0.007
Low-density lipoprotein cholesterol	-0.193	0.002
Triglycerides	-0.033	0.601
HbA1c	0.125	0.065
Albumin	-0.382	<0.0001
Estimated GFR	-0.653	<0.0001
High sensitivity C-reactive protein	0.303	<0.0001
White blood cell count	0.407	<0.0001
Visfatin	0.274	0.030
Adiponectin	0.419	<0.0001
Fatty acid-binding protein 4	0.769	<0.0001
**ECG parameters**		
Heart Rate	0.359	<0.0001
PR interval	0.175	0.007
QRS duration	0.119	0.061
QT interval	0.003	0.964
QTc interval	0.359	<0.0001
**Echocardiographic parameters**		
Aortic root diameter	0.100	0.213
Left atrial diameter	0.176	0.017
Left ventricular mass index	0.205	0.012
Interventricular septum thickness at end-diastole	0.138	0.048
Left ventricular internal dimension at end-diastole	0.123	0.136
Left ventricular posterior wall thickness at end-diastole	0.143	0.041
End-diastolic volume	0.110	0.194
End-diastolic volume index	0.121	0.151
End-systolic volume	0.202	0.010
End-systolic volume index	0.234	0.003
Left ventricular internal dimension at end-systole	0.179	0.016
Stroke volume	-0.002	0.982
Fractional shortening	-0.222	0.001
Ejection fraction	-0.225	0.002
Interventricular septum/Left ventricular posterior wall	-0.129	0.428
Ratio of E to A	0.031	0.715
Peak E-wave velocity	-0.166	0.333
Peak A-wave velocity	0.030	0.856
Peak velocity	0.056	0.496
Ratio of the left atrial dimension to the aortic annulus dimension	0.041	0.556

GFR: glomerular filtration rate; QTc: corrected QT.

**Table 3:** Multiple linear regression analysis for fatty acid-binding protein 3, serum biomarkers, ECG parameters, and echocardiographic parameters

Parameter	Fatty acid-binding protein 3	
	β*	*p*-value*
**Serum biomarkers**		
Total cholesterol	-0.068	0.268
High-density lipoprotein cholesterol	-0.121	0.049
Low-density lipoprotein cholesterol	-0.067	0.284
Triglycerides	-0.028	0.643
High sensitivity C-reactive protein	0.246	<0.0001
White blood cell count	0.394	<0.0001
Visfatin	0.213	0.044
Adiponectin	0.360	<0.0001
Fatty acid-binding protein 4	0.705	<0.0001
**ECG parameters**		
Heart Rate	0.283	<0.0001
PR interval	0.079	0.209
QRS duration	0.083	0.158
QT interval	-0.021	0.719
Corrected QT interval	0.256	<0.0001
**Echocardiographic parameters**		
Aortic root diameter	0.066	0.419
Left atrial diameter	0.183	0.011
Left ventricular mass index	0.151	0.048
Interventricular septum thickness at end-diastole	0.082	0.236
Left ventricular internal dimension at end-diastole	0.114	0.149
Left ventricular posterior wall thickness at end-diastole	0.094	0.175
End-diastolic volume	0.105	0.193
End-diastolic volume index	0.093	0.244
End-systolic volume	0.161	0.024
End-systolic volume index	0.168	0.022
Left ventricular internal dimension at end-systole	0.134	0.051
Stroke volume	0.041	0.574
Fractional shortening	-0.157	0.017
Ejection fraction	-0.159	0.018
Interventricular septum/Left ventricular posterior wall	-0.180	0.213
Ratio of E to A	0.055	0.502
Peak E-wave velocity	-0.035	0.806
Peak A-wave velocity	-0.105	0.437
Peak velocity	0.012	0.886
Ratio of the left atrial dimension to the aortic annulus dimension	0.069	0.296

*Adjusted for age, sex, body mass index, diabetes mellitus and hypertension by multiple linear regression analysis.

**Table 4:** Baseline characteristics of the study population stratified by category of QTc prolongation status at baseline*

Variable	Normal	Borderline	Abnormal	*P*-value
No	119	51	79	
Sex (male/female)	88/31	42/9	55/24	0.266
Age (years)	68.4±11.3	73.7±11.0	74.1±10.6	0.001
Age range	42-99	51-93	40-94	
Body mass index (kg/m^2^)	25.7±3.7	25.6±3.9	25.7±4.3	0.988
Waist (cm)	91.6±9.9	93.0±8.9	92.2±11.3	0.760
Hypertension (n, %)	91 (76.5)	42 (82.4)	65 (82.3)	0.522
Hyperlipidemia (n, %)	85 (71.4)	32 (62.8)	49 (62.0)	0.311
Diabetes mellitus (n, %)	45 (37.8)	24 (47.1)	50 (63.3)	0.002
LVSD (n, %)	10 (8.4)	9 (17.7)	22 (27.9)	0.013
Current smoking (n, %)	46 (38.7)	22 (43.1)	30 (38.0)	0.765
Corrected QT interval (ms)	413±19	445±9	484±26	<0.0001
Systolic blood pressure (mmHg)	134±19	133±25	135±24	0.836
Diastolic blood pressure (mmHg)	76±12	76±10	75±13	0.884
Sodium (mEq/L)	139.1±3.7	140.2±4.5	138.3±3.9	0.026
Potassium (mEq/L)	3.8±0.4	3.8±0.5	3.9±1.0	0.661
Calcium (mg/dl)	8.7±0.9	8.6±1.1	8.6±0.9	0.736
Fasting glucose (mg/dl)	129.4±54.0	127.7±40.6	160.2±80.9	0.001
HbA1c (%)	6.8±1.5	6.8±1.2	7.2±1.6	0.280
T-cholesterol (mg/dl)	175.6±43.5	168.9±34.9	164.8±40.7	0.183
Triglyceride (mg/dl)	117.0 (84.0-175.0)	115.0 (86.0-179.0)	115.0 (86.0-164.0)	0.664
HDL-cholesterol (mg/dl)	40.7±15.3	39.9±10.5	38.0±11.6	0.375
LDL-cholesterol (mg/dl)	108.0±40.8	99.7±27.6	94.6±31.8	0.036
Uric acid (mg/dl)	6.4±1.5	6.9±3.3	7.1±2.0	0.098
Blood urea nitrogen (mg/dl)	19.3±10.6	23.4±10.9	26.7±16.5	0.001
Creatinine (mg/dl)	1.2 (1.1-1.4)	1.3 (1.1-1.5)	1.4 (1.2-1.8)	0.003
Albumin (g/dl)	4.1±0.4	3.9±0.5	3.8±0.4	0.0002
Hematocrit (%)	40.1±4.3	39.5±5.0	37.4±6.6	0.002
Hemoglobin (g/dl)	13.4±1.7	13.2±1.9	12.4±2.4	0.002
Estimated GFR (ml/min/1.73m^2^)	61.0±17.5	55.0±18.6	47.4±22.5	<0.0001
Fatty acid-binding protein 3 (ng/mL)	1.0 (0.8-1.6)	1.2 (0.9-3.6)	2.6 (1.3-10.2)	<0.0001
Fatty acid-binding protein 4 (ng/mL)	9.1 (5.0-13.6)	15.9 (9.3-27.0)	17.8 (9.9-49.7)	0.003
Troponin-I (ng/mL)	0.1 (0.0-0.4)	0.1 (0.0-0.4)	0.1 (0.0-0.3)	0.376
Hs-CRP (mg/L)	1.5 (0.8-4.4)	2.2 (0.8-7.4)	4.4 (1.1-10.2)	0.012
Adiponectin (μg/mL)	3.7 (1.9-7.4)	4.0 (1.6-8.8)	7.4 (3.1-14.7)	0.007
White blood cell count (×10^9^/L)	6.907±1.895	7.032±2.354	8.037±3.171	0.005
No. of diseased coronary arteries	1.9±1.1	2.3±0.9	2.2±0.9	0.061
Gensini score	30.0 (14.6-65.1)	51.0 (17.0-86.0)	41.5 (18.0-83.0)	0.713
Number of stent	0 (0-1)	0 (0-1)	0 (0-0.0)	0.065
Anti-arrhythmic medication (n, %)	7 (5.9)	4 (7.8)	9 (11.4)	0.376
Beta-blockers (n, %)	54 (45.4)	21 (41.2)	24 (30.4)	0.105
Diuretics (n, %)	24 (20.2)	16 (31.4)	26 (32.9)	0.094
Statins (n, %)	65 (54.6)	26 (51.0)	30 (38.0)	0.067

Data are expressed as mean ± SD, number (percentage), or median (interquartile range). LVSD, left ventricular systolic dysfunction; HDL, high-density lipoprotein; LDL, low-density lipoprotein; GFR, glomerular filtration rate; Hs-CRP, high-sensitivity C-reactive protein. *Classification of QTc prolongation: normal men ≤ 430 ms; women ≤450 ms; borderline men 431-450 ms; women 451-470 ms; abnormal men ≥451 ms; women ≥471 ms.

**Table 5:** Demographic and clinical characteristics according to tertiles of fatty acid-binding protein 3

Parameter	Low FABP3 ≤1 ng/mL	Medium FABP3 = 1.1-2.1 ng/mL	High FABP3 >2.1 ng/mL	*p*-value
No.	81	85	83	
Sex (male/female)	66/15	61/24	58/25	0.190
Age (years)	63.7±9.7	74.4±9.2	75.4±11.2	<0.0001
Hypertension (n, %)	55 (67.9)	72 (84.7)	71 (85.5)	0.007
Diabetes mellitus (n, %)	24 (29.6)	39 (45.9)	56 (67.5)	<0.0001
Hyperlipidemia (n, %)	55 (67.9)	62 (72.9)	49 (59.0)	0.154
Abnormal QTc interval (n, %)	12 (14.8)	23 (27.1)	44 (53.0)	<0.0001
LVSD (n, %)	8 (9.9)	12 (14.1)	20 (24.1)	0.043
Current smoker (n, %)	28 (34.6)	35 (41.2)	35 (42.7)	0.529
No. of diseased coronary arteries	1.9±0.9	2.1±1.0	2.2±0.9	0.053
Number of stents	0 (0-1)	0 (0-1)	0 (0-1)	0.693
Body mass index (kg/m^2^)	25.2±3.4	26.0±4.2	25.9±4.2	0.375
Systolic BP (mmHg)	129±17	138±20	136±26	0.019
Diastolic BP (mmHg)	75±10	77±10	76±15	0.779
Total-cholesterol (mg/dl)	179.7±45.1	169.9±36.0	163.0±40.8	0.034
Triglycerides (mg/dl)	119.0 (84.5-180.0)	112.0 (86.0-162.5)	115.0 (85.5-172.0)	0.867
HDL-cholesterol (mg/dl)	40.2±9.9	41.1±16.2	37.7±12.8	0.238
LDL-cholesterol (mg/dl)	113.4±40.9	99.0±32.1	94.0±32.1	0.002
Fasting glucose (mg/dl)	122.8±39.9	131.9±58.8	161.6±78.3	0.0002
HbA1c (%)	6.6±1.2	7.0±1.6	7.2±1.5	0.055
Albumin (g/dl)	4.2±0.3	4.0±0.4	3.7±0.5	<0.0001
Estimated GFR (ml/min/1.73 m^2^)	69.7±14.2	57.3±14.5	39.6±19.3	<0.0001
Hs-CRP (mg/L)	1.3 (0.8-3.0)	2.1 (1.1-7.3)	6.9 (1.7-15.7)	0.0001
White blood cell count (×10^9^/L)	6.674±1.553	6.679±1.856	8.520±3.276	<0.0001
Visfatin (ng/mL)	9.5 (7.1-12.5)	10.9 (7.0-18.8)	14.6 (9.6-31.3)	0.042
Adiponectin (μg/mL)	1.6 (0.9-2.9)	2.8 (1.9-5.6)	5.4 (1.0-8.6)	<0.0001
FABP4 (ng/mL)	5.4 (4.4-8.9)	12.8 (8.4-16.1)	42.5 (18.3-66.1)	<0.0001
ECG parameters				
Heart Rate (bpm)	70.0±13.3	70.9±14.0	84.1±20.3	<0.0001
PR interval (ms)	163.8±23.5	171.8±31.1	177.3±38.6	0.029
QRS duration (ms)	93.8±15.6	96.4±19.1	99.8±20.6	0.114
QT interval (ms)	392.6±60.5	405.0±56.7	395.3±51.0	0.322
QTc interval (ms)	427.4±31.1	439.4±31.7	458.5±41.1	<0.0001
Echocardiographic parameters				
Aortic root diameter (cm)	3.1±0.5	3.3±0.5	3.2±0.5	0.088
Left atrial diameter (cm)	3.7±0.6	3.8±0.6	4.0±0.7	0.039
LVMI (g/m^2^)	114.3±27.6	128.1±42.6	133.9±49.7	0.047
IVSd (cm)	1.1±0.2	1.2±0.2	1.2±0.2	0.006
LVIDd (cm)	4.8±0.5	4.9±0.7	5.0±0.8	0.384
LVPWd (cm)	1.0±0.2	1.1±0.2	1.1±0.2	0.027
End-diastolic volume (ml)	111.8±28.2	115.7±44.0	122.8±47.3	0.421
End-diastolic volume index (ml/m^2^)	64.7±17.1	65.9±26.7	70.6±28.2	0.463
End-systolic volume (ml)	39.9±19.5	50.7±35.7	57.1±38.6	0.025
End-systolic volume index (ml/m^2^)	23.2±12.9	27.6±20.2	32.7±21.5	0.037
LVIDs (mm)	3.1±0.6	3.4±0.9	3.5±1.0	0.029
Stroke volume (ml)	70.1±20.6	67.3±20.2	68.0±21.7	0.764
Fractional shortening (%)	37.0±7.1	33.5±8.8	31.9±9.0	0.002
Ejection fraction (%)	66.1±10.1	61.3±12.9	58.3±13.6	0.002
IVS/LVPW	1.1±0.2	1.1±0.1	1.1±0.1	0.645
Ratio of E to A	1.0±0.3	1.0±0.6	1.1±0.5	0.474
Peak E-wave velocity (cm/s)	65.4±14.8	67.1±20.7	63.7±19.1	0.921
Peak A-wave velocity (cm/s)	73.1±20.9	82.8±23.7	71.1±25.1	0.413
Peak velocity (cm/s)	227.0±50.2	226.7±43.9	233.4±78.8	0.817
LA/AO	1.2±0.2	1.2±0.3	1.2±0.3	0.493
MACEs (n, %)	29 (35.8)	39 (45.9)	41 (49.4)	0.191
All-cause mortality (n, %)	1 (1.2)	6 (7.1)	17 (20.5)	<0.0001

Data are expressed as mean ± SD, or number (percentage). LVSD, left ventricular systolic dysfunction; BP, blood pressure; HDL, high-density lipoprotein cholesterol; LDL, low-density lipoprotein cholesterol; GFR, glomerular filtration rate; Hs-CRP, high sensitivity C-reactive protein; FABP, fatty acid-binding protein; QTc, corrected QT; LVMI, left ventricular mass index; IVSd, interventricular septum thickness at end-diastole; LVIDd, left ventricular internal dimension at end-diastole, LVPWd, left ventricular posterior wall thickness at end-diastole; LVIDs, Left ventricular internal dimension at end-systole; IVS/LVPW, interventricular septum/Left ventricular posterior wall; LA/AO, ratio of the left atrial dimension to the aortic annulus dimension; MACEs, major adverse cardiovascular events. Abnormal QTc interval defined as men ≥451 ms; women ≥471 ms; Left ventricular systolic dysfunction defined as left ventricular ejection fraction ≤50%.
