# Supplementary material for: Elevated plasma fatty acid-binding protein 3 is related to prolonged corrected QT interval and reduced ejection fraction in patients with stable angina
Source: Int J Med Sci. 2021 Mar 11;18(9):2076–85. doi: 10.7150/ijms.54508 (PMC8040394; doi:10.7150/ijms.54508)
Supplement: Supplementary file 1 — Supplementary materials. [file ijmsv18p2076s1.pdf]

Supplementary material for the paper titled "Elevated plasma fatty acid-binding protein 3 is related to prolonged corrected QT interval and reduced ejection fraction in patients with stable angina"

Yung-Chuan Lu[1,8], Thung-Lip Lee[2,8], Chin-Feng Hsuan[2,6,12], Wei-Chin Hung[2,6], Cheng-Ching Wu[2,7,11], Chao-Ping Wang[2,8], Ching-Ting Wei[3,8,9,10], Teng-Hung Yu[2,7], Fu-Mei Chung[2], Yau-Jiunn Lee[5], and I-Ting Tsai[4,6,*]

[1]Division of Endocrinology and Metabolism, [2]Division of Cardiology, Department of Internal Medicine, and [3]Division of General Surgery, Department of Surgery, and [4]Department of Emergency, E-Da Hospital, Kaohsiung, 82445 Taiwan

[5]Lee's Endocrinology Clinic, Pingtung, 90000 Taiwan

[6]School of Medicine, and [7]The School of Chinese Medicine for Post Baccalaureate, and [8]School of Medicine for International Students, College of Medicine, and [9]Department of Biomedical Engineering, and [10]Department of Electrical Engineering, I-Shou University, Kaohsiung, 82445 Taiwan

[11]Division of Cardiology, Department of Internal Medicine, E-Da Cancer Hospital, Kaohsiung 82445 Taiwan

[12]Division of Cardiology, Department of Internal Medicine, E-Da Dachang Hospital, Kaohsiung, Taiwan

Electrocardiogram, QT and QTc interval measurements: QT interval was defined as the interval between the first deflection of the QRS complex and the end of the T wave. The end of the T wave was determined by extending a tangent from the steepest portion of the downslope of the T wave until it crossed the T-P segment. The QT and RR intervals were averaged over three consecutive complexes in lead II in sinus rhythm. During other rhythms, QT and RR intervals were averaged over all complexes on the 10-second lead II rhythm strip on the 12-lead ECG. The QTc intervals were calculated using Bazett's formula ($QTc=QT/\sqrt{RR}$) [23,24]. In brief, the ECG tracings were first analyzed by two independent cardiologists and the senior supervising cardiologist who were blinded to the participants' demographics. Inter-reader discrepancies were resolved by direct comparison and adjudicated by the supervising cardiologist. If the T wave amplitude was too flat so that the end of the wave could not be identified or if differences between the QTc measurements between the two independent cardiologist were too great so that they could not be resolved by the supervisor, the data were excluded from the study. The final QTc value was the average of QTc values calculated by the supervisor and the two blinded independent cardiologists. Inter-reader reproducibility assessments for the QT measurements showed a coefficient of reliability of 0.995, and a Pearson's correlation coefficient of 0.995. Comparing inter-reader QT measurements using the paired *t*-test did not achieve statistical significance (P=0.35). In addition, extremely rapid (>150 bpm) and extremely slow (<40 bpm) heart rate recordings were also excluded to eliminate the influence of heart rate on QT measurements [25,26]. In this study, QTc prolongation was categorized into three sex-specific categories based on the opinion of an ad hoc group according to the latest European regulatory guidelines and a previous study [27]. The cutoff points were ≤450 ms (normal), 451 to 470 ms (borderline), and >470 ms (prolonged) in women, and ≤430 ms (normal), 431 to 450 ms (borderline), and >450 ms (prolonged) in men.

Echocardiographic examination: The echocardiography examinations were performed during a resting period in the morning and in the left lateral position, using a Vivid 7 System (GE Healthcare, General Electric Company, Wauwatosa, USA) with 3-7 MHz transducers and M-mode, two-dimensional and Doppler (pulsed, continuous, color and tissue) echo modalities. Examinations for the long and short parasternal and apical axes, as well as for 2, 3, 4 and 5-chamber views were performed. The cardiac structure and function were assessed using M-mode guided by two-dimensional imaging to obtain the following variables: aortic root end-diastolic diameter; left atrial end-systolic anteroposterior diameter; end diastolic interventricular septal thickness; end diastolic left ventricular posterior wall thickness; left ventricular end-diastolic diameter and volume, and left ventricular end-systolic diameter and volume.

Left atrial (LA) dilatation was defined in the presence of an LA anteroposterior diameter >4.0 cm, and left ventricular (LV) dilatation was defined when the LV diastolic diameter was >5.4 cm. LV ejection fraction was calculated from the apical 4-chamber view using the modified Simpson method, and left ventricular systolic dysfunction was defined as an LV ejection fraction ≤50% [30]. LV mass was calculated using a 2-dimensional method and indexed to the body surface area. Mitral flow was assessed in the apical 4-chamber view using pulsed Doppler. The sample was positioned between the distal extremities of the mitral valve leaflets and then the following variables were obtained: early (E) and late diastolic mitral velocities (A) and E/A ratio.
